# End User and Primary Care Physicians’ Perspectives on Digital Innovations in Dementia Risk Detection: Focus on a Digital Sleep Biomarker

**DOI:** 10.2196/74307

**Published:** 2025-12-01

**Authors:** Ríona Mc Ardle, Marie Poole, Sophie Horrocks, Josh King-Robson, Jonathan M Schott, David Sharp, Matthew Harrison, Louise Robinson

**Affiliations:** 1 Translational and Clinical Research Institute Newcastle University Newcastle Upon Tyne United Kingdom; 2 NIHR Newcastle Biomedical Research Centre Newcastle Upon Tyne United Kingdom; 3 Population Health Sciences Institute Newcastle University Newcastle Upon Tyne United Kingdom; 4 Care Research and Technology Centre UK Dementia Research Institute London United Kingdom; 5 Institute of Global Health Innovation Helix Centre Imperial College London London United Kingdom; 6 Queen Square Institute of Neurology Dementia Research Centre University College London London United Kingdom; 7 University College London UK Dementia Research Institute London, England United Kingdom; 8 Department of Brain Sciences, Faculty of Medicine Imperial College London London United Kingdom

**Keywords:** dementia, digital technology, digital health, mass screening, qualitative research, community-based participatory research

## Abstract

**Background:**

Dementia is a global health priority. Early identification in asymptomatic or mildly symptomatic individuals (ie, dementia risk detection) is proposed as a clinical solution for early intervention and could support researchers to identify novel neuropathological targets and recruit to clinical trials. Digital biomarkers of behavioral or physiological markers, including sleep, are cited as a potential low-cost, noninvasive, and objective method for dementia risk detection. Understanding perspectives on digital biomarkers, particularly acceptability, from potential end users and clinical staff is required when considering implementation within any clinical service. With emerging evidence of sleep as a risk marker for dementia, the efficacy of the Dementia Research Institute Sleep Index (DRI-SI), based on continuous remote monitoring of sleep patterns detected by a digital sleep mat, for dementia risk detection, is currently being explored by the InSleep46 study.

**Objective:**

This qualitative substudy aimed to explore perspectives of potential end users and primary care physicians regarding the use of a digital sleep mat to measure the DRI-SI and its application towards dementia risk detection.

**Methods:**

Thirty-one potential end users (age: 31-82 years, 11 female and 20 male) from Newcastle and London, United Kingdom, with personal or caregiving experience related to dementia, participated in qualitative focus group workshops. They shared opinions on integrating the sleep mat into their homes, the DRI-SI’s potential for identifying dementia risk, and the necessary information for engagement with related clinical services. Seven primary care physicians from across England participated in semistructured interviews regarding the potential application of the DRI-SI in dementia risk detection and its integration into current clinical practice. Inductive thematic analysis was conducted to identify key themes.

**Results:**

Four key themes emerged from end user focus groups: (1) practical use, (2) prospective acceptability, (3) clinical management, and (4) data concerns. Three main themes came from the semistructured interviews with physicians: (1) prospective acceptability, (2) health care provision, and (3) practical considerations. Common themes were identified in both groups but held differing perspectives. End users were focused on practical aspects of integrating the digital sleep mat within their daily life, the effect of the DRI-SI on clinical care, and privacy concerns regarding data use. Primary care physicians were concerned more broadly with how the DRI-SI and dementia risk detection service would integrate into current clinical practice, the impact on clinical resources and patient well-being, and the need for clinical actionability and guidance on discussing results with patients.

**Conclusions:**

End users would find the DRI-SI acceptable as part of their clinical care, but primary care physicians require a more robust evidence base. Future research should explore the integration of the DRI-SI into clinical care/research pathways to enhance clinical acceptability. Five key recommendations have been made for further development of digital biomarkers for dementia risk populations.

## Introduction

Dementia is considered a global health priority by the World Health Organization and the leading cause of disability and dependency amongst older adults [1]. Dementia is a syndrome characterized by significant cognitive impairment leading to impairments in functional abilities. Fifty-five million people are currently living with the condition worldwide, with numbers expected to rise to 139million by 2050 [2]. Dementia is not an inevitability; the recent Lancet Commission suggested that ≈45% of all dementia cases are preventable, reporting 14 known modifiable risk factors [3]. Further risk factors, such as sleep, are under investigation [4]. In the absence of a globally accessible cure or treatment, the World Alzheimer’s Report 2023 declared dementia risk reduction to be the most proactive and feasible way to combat dementia [2].

Dementia risk detection, which involves screening for dementia-causing diseases in those who are apparently well, has been proposed to enhance identification of high-risk individuals in asymptomatic populations [5-8]. For patients, this would provide access to earlier treatments, such as personalized risk reduction interventions [6], or disease-modifying therapies for Alzheimer disease; shown to be most efficacious when given early in the disease course [9]. From a scientific perspective, dementia risk detection may support identification of neurodegenerative changes in asymptomatic individuals, providing novel disease-modifying targets, and aiding recruitment of high-risk individuals into clinical trials [10]. There is a growing interest in “digital biomarkers,” defined as objective, quantifiable physiological and behavioral data measured using digital technologies, to act as low-cost, noninvasive methods to detect dementia risk [5,10]. Digital sleep biomarkers may be one such method [5,11].

Sleep disturbances are a common symptom of dementia, presenting as difficulties falling or remaining asleep, excessive daytime napping, “sundowning” whereby people become confused and agitated during evenings, and rapid eye movement sleep behavior disorder [12-15]. Although the cause-and-effect process is unclear, sleep disturbances may occur prior to cognitive symptom onset as they are associated with an increased risk of developing dementia in both cognitively normal older adults and people with Parkinson disease [16,17]. Early evidence indicates that differences in sleep patterns, quantified by a digital sleep biomarker, known as the Dementia Research Institute Sleep Index (DRI-SI), may be able to distinguish dementia from normal ageing [11]. The DRI-SI can be objectively measured through continuous remote monitoring of sleep patterns using an under-mattress digital sleep mat (Withings Sleep analyzer) and is now being explored in asymptomatic individuals, with the view of assessing its utility to detect dementia risk prior to cognitive symptom onset.

This research is timely, given the rising interest in a “digital first” health care system in England. Most recently, the UK’s Digital Health and Social Care Plan set out government-level goals to improve patient access to health care services via digital means and expand remote monitoring to manage long-term conditions [18]. National and international policies, coupled with significant international efforts to technically and clinically validate digital biomarkers and endpoints, indicate that digital dementia risk detection tools may soon be a feasible and useful avenue to support dementia risk reduction by identifying high-risk individuals requiring intervention [4,5,19-24]. However, such tools and resulting digital risk scores must be acceptable to both the potential end users (ie, the general public) and clinicians in order to be commissioned within health care services [8,25], especially primary care, where most screening occurs [26]. Recent surveys of the public, including underserved groups, have indicated that dementia risk detection is generally acceptable to the public [27]. However, there has been limited exploration regarding their perspectives on the use of a continuous remote monitoring digital device, such as a digital sleep mat, for this purpose [27,28]. A recent systematic review has indicated that primary care physicians have mixed views on the implementation of dementia risk screening but are interested in engaging in dementia risk reduction approaches [29]. Negative perspectives on dementia risk screening were largely attributed to limited time, resources, and clinicians’ lack of education and knowledge in the area. Conversely, the review highlighted positive perceptions of a dedicated dementia screening service that would provide protected time and resources to conduct screening and communicate results with patients in a nonstigmatizing manner. Regarding digital biomarkers, one study has reported that primary care physicians recognize the potential of wearable technology to detect dementia but hold concerns regarding health inequities caused by direct-to-consumer products [30]. As such, there is currently not enough literature on this topic to infer how potential end users and primary care physicians would perceive a digital sleep mat and the DRI-SI, and their use within a potential dementia screening service. Further research is therefore required to identify prospective barriers to acceptability and to proactively consider solutions prior to resource-intensive development of the DRI-SI, ensuring that issues around appropriateness and uptake are addressed from the outset. The aim of this study is to understand the perspectives of (1) potential end users and (2) primary care physicians regarding the use of a digital sleep mat for dementia risk detection.

## Methods

### Overview

This study is part of a larger study (InSleep46: Detecting and monitoring dementia using dynamic digital biomarkers of night-time behavior and sleep) with the overall aim to produce a digital biomarker, the DRI-SI, for use in screening for neurodegenerative pathology and progression towards dementia [11]. The purpose of the qualitative substudy was to consult the public and relevant professionals on the acceptability and practicality of using digital sleep data as part of a hypothetical dementia screening service, whereby a digital sleep mat would be supplied to participants’ homes for 3 months, and the DRI-SI would be derived and communicated. An exploratory, health services research approach was adopted, using qualitative methods, to explore the perspectives of potential end users, including those who were worried about their dementia risk, with a family history or caregiver experience of dementia, and physicians regarding the potential implementation of the digital sleep mat for dementia risk detection [31]. This approach studies how health care services are organized, delivered, and experienced by multiple stakeholders, and seeks to understand how these interactions affect access and outcomes of care.

### Ethical Considerations

The study was approved by the Imperial College Ethics Committee (reference number: 6554509) on May 15, 2023. Participants were provided with written information about the study, and their written informed consent was obtained prior to participation. Each participant received remuneration in the form of either shopping vouchers or financial payment in line with National Institute for Health Research (NIHR) guidance for their involvement after participation [32]. No identifiable information about participants is provided.

### Recruitment

Potential end users were recruited via local research and community networks in the North East of England and London using purposeful sampling, particularly advertising through organizations inclusive to rural and low socioeconomic communities [33]. The inclusion criteria for potential end users were as follows: experience of dementia diagnosis from perspective of a family caregiver, professional role, or recent experience of own diagnosis; or at risk of developing dementia, or aged 65 years, or worried about their own memory or cognitive abilities; able to communicate effectively in English; resident in the United Kingdom; physically able to attend and participate in an in-person workshop or having the digital skills and equipment to join a web-based group meeting (Zoom/Teams Call). Potential participants were excluded from the study if they had moderate/advanced dementia or if they lacked capacity to understand the project and consent to involvement in line with the principles set out in the Mental Capacity Act (2005) Code of Practice [34].

This study was also designed to capture clinical perspectives; during end user interviews (ie, general public), participants highlighted that the most appropriate clinical context for implementation would be primary care. We therefore focused our recruitment on primary care physicians to ensure that the clinical perspectives reflected where the tool might be applied in practice. Primary care physicians were recruited using convenience and snowball sampling from across England [35]. All physicians worked in the National Health Service (NHS) in England as general practitioners, with experience of providing health care to people with suspected cognitive impairment and dementia. Physicians were included if they had experience in dementia diagnosis in a professional role, were residents in the United Kingdom, and could communicate effectively in English.

### Data Collection

Data collection was conducted with potential end service users prior to exploring physician views to allow us to structure physician interviews based on the emergent findings from focus groups with end service users.

Four in-person focus groups with potential end users took place between September 2023 and January 2024, two in Newcastle and 2 in London, facilitated by 2 experienced (MP and LR) and 3 novice qualitative researchers (SH, MH, and RMA; 4 female and 1 male).

Before beginning the focus group discussion, the moderators outlined the InSleep46 study to participants, including the associations between sleep and dementia risk, the practical use of the sleep mat, an example of the data received from the sleep mat, and a brief explanation of the DRI-SI. A semistructured topic guide was developed to explore initial perceptions of the sleep mat, probing on practicalities of use and trust in the data; willingness to use the proposed screening service and DRI-SI to identify dementia risk, probing on using the sleep mat for 3 months and receiving feedback following this, and the information provided when receiving the DRI-SI score; and where participants envisioned this service sitting within health care or as the consumer-based landscape (Multimedia Appendix 1). The topic guide evolved iteratively during data gathering through discussions within the research team. All focus groups were audio recorded and transcribed verbatim using a university-approved transcription company, then checked and anonymized by the research team (RMA).

A semistructured interview approach was used with physicians (Multimedia Appendix 2 for interview schedule). Key themes and considerations identified in the end user focus groups were embedded into an interview schedule, which guided questioning, with ad hoc follow-up questions used to explore salient points. A total of 6 of 7 interviews were conducted by one of 2 researchers (MP and RMA) via one-to-one teleconferencing call, while one interview was in person. Informed consent was obtained in writing prior to the interview and verbally recorded at the beginning of the interview. All interviews were audio recorded, transcribed verbatim, checked, and anonymized by the research team (RMA) prior to analysis.

### Public Patient Involvement

Two contributors with lived experience of dementia were involved in this substudy. One contributor supported the development of the aims and methodology at the grant proposal stage, before withdrawing due to changes in their condition. The second contributor has been involved in the conduct of the research, including but not limited to reviewing interview schedules, supporting recruitment efforts, attending project meetings, and contributing to the thematic analysis process and interpretation of data, by providing insights from their lived experience.

### Data Analysis

All transcripts were analyzed using inductive thematic analysis, aligned with a critical realism epistemology [36]. NVivo (version 14; Lumivero) software was used for the management of transcripts [37]. This systematic, “bottom-up,” reflexive approach involved multiple phases, and data collection and analysis ran in parallel. A member of the research team (RMA) familiarized herself with the data through repeated reading of transcripts. She generated initial codes and discussed these with the research team, including all focus group moderators (MP, MH, SH, and LR) and a Public Patient Contributor with lived experience of dementia, using triangulation to ensure a unified interpretation and relevance to study aims. First-order themes were constructed through researchers’ interpretation of the coded data; these were reviewed regularly in discussions with the research team and refined further, ensuring credibility and consistency in analysis and minimizing bias in interpretation. First-order themes were clustered into a second-order series of themes based on commonality of meaning and patterns in the data. This was further refined, with second-order themes expanding to encompass others or shrinking for specificity. Themes were reviewed against the coded data and the entire dataset (either end users or primary care physicians, where relevant) to ensure participants’ perspectives were accurately captured.

### Reflexivity

The research team was multidisciplinary, including expertise in dementia research, qualitative methods, human-centered design, digital health, and primary care practice. While the team collaborated with the wider InSleep46 team, this work package operated independently from the team developing the DRI-SI. The development of the end user topic guide was developed prior to the first author (RMA) joining the team, although she observed all end user focus groups and kept field notes, using these to input into the interview schedule for primary care physicians, which she conducted. She led the analysis on both sets of data. The first author’s expertise is in digital health and dementia research, with prior involvement in dementia risk detection research; this may have initially shaped expectations around the perceived acceptability and value of the DRI-SI. Reflexive engagement was maintained throughout analysis through memoing, maintaining, and iteratively refining a codebook, and regular team discussions. The codebook evolved from an initially broad set of descriptive codes to a refined structure that captured the conceptual relationships between themes. Discussions with other team members were used to challenge assumptions, refine theme definitions, and ensure interpretations remained grounded in participants’ accounts rather than disciplinary biases.

## Results

### Participants

Thirty-one potential end user participants (age: 31-82 years; 11 females and 20 males; 25/31, 81% White) from Newcastle and London, United Kingdom, with personal or caring experience related to dementia, took part in focus group workshops. Seven primary care physicians were recruited from across England and had a range of clinical experience and related interests, outlined in Table 1.

**Table 1:** Demographic information on primary care physicians.

Participant	Sex	Practice area	Common types of patients	Additional expertise
GP1	Male	Two practices: Urban, suburban; Newcastle, North East	University students Older patients Families Care home residents	Academic researcher in dementia risk
GP2	Female	Two practices: Urban, suburban; Newcastle, North East	Care home residents Dementia/memory problems	Technology Lead for Clinical Commissioning Group (CCG) previously
GP3	Female	Two practices: Urban, suburban; Newcastle, North East	University students Wide age range Dementia risk cases	—^a^
GP4	Male	Semirural, Northumberland, North East	Older patients Care home residents Lower risk profile than other areas	Researcher focused on chronic illness in care homes
GP5	Male	Urban, London, South East	Older patients with cognitive issues/dementia Frailty management Nursing home residents	Researcher in technology and dementia
GP6	Male	Semirural, Bristol, South West	Patients with dementia Farming communities	Researcher in dementia diagnostic tests
GP7	Female	Urban, Manchester, North West	Patients with memory concerns and dementia Health disparities	Researcher on health inequity in dementia and primary care

^a^Not available.

Briefly, four main themes were constructed through research interpretation from the end user focus groups: (1) practical use, (2) prospective acceptability, (3) clinical management, and (4) data concerns. Three main themes were generated from analysis of the semistructured interviews with primary care physicians: (1) prospective acceptability, (2) health care provision, and (3) practical considerations. Although common themes were identified for both groups concerning prospective acceptability, practical considerations, and health care management, the groups approached these from different perspectives. End users were primarily concerned about the practicalities of using the digital sleep mat and had privacy concerns about personal data. Primary care physicians wanted to know how the device and corresponding DRI-SI would fit into broader health care delivery and decision-making processes. Tables 2 and 3 provide definitions of themes and subthemes, with examples of common topics, for each stakeholder group, respectively. Thematic analysis results are presented below for end users, followed by the primary care physicians.

**Table 2:** Themes and subthemes which were generated from focus groups with potential end users.

Theme and subtheme			Definition		Common topics
**Practical use:** **considers every day, tangible aspects of using the sleep mat**					
	Considerations for practical use	Refers to the practicalities of using the sleep mat, which an end user may consider an issue or barrier for use.		Bed features Design features Digital connectivity Out-of-home activities Reminders/prompts Safety considerations Technical support Time period for device use	
	Cost implications	Refers to the costs associated with the sleep mat and proposed dementia screening services, whether experienced by end users, clinicians, or the National Health Service.		End user costs Cost-benefit analysis Supplementation of costs	
**Prospective acceptability:** **focuses on perceptions and attitudes of end users regarding the sleep mat**					
	General acceptability of device and digital technology	Perception of acceptable components or uses of the sleep mat and digital technology.		Positive perceptions Negative perceptions Use of technology Data monitoring Trust in commercial or third-party organizations Trust in data access Trust in technology	
	Comparison to other digital products	Perceptions of the sleep mat to other digital devices, such as smart watches, or experiences of using similar devices to the sleep mat, that is, devices which assess sleep.		Competition with existing devices Information provision	
	Comparison to other clinical markers	Perceptions of how the sleep mat compares to other clinical markers for dementia or health (eg, blood biomarkers).		Part of the clinical toolkit Preferential perception of the sleep mat Time period	
**Clinical management:** **considers the integration of the sleep mat into the end users’ health care journey**					
	Service provision	Considers who the dementia risk detection service should be provided to and at what clinical timepoint and stage.		Early detection Memory impairments Prevention tool Sleep problems Population of interest Concerns with sleep Safety device General health and well-being Monitoring disease progression	
	Reciprocal communication	Two-way communication of information relating to the sleep mat/service (eg, results) from both the device itself or from clinical services.		Clinical service Communication of data to the patient Communication of results Patient to clinician communication	
	Postscreening actions	Actions that would be taken following results from the sleep mat/clinical service.		Clinical support Self-management	
**Data concerns:considers data handling, access, and accuracy**					
	Data accuracy	Experiences or confounding factors that may impact the accuracy of the sleep data.		Accessibility Alcohol Bed features Bed partners Erratic sleep patterns Medication Mental Health Microphone disturbances Nonsleep bed behaviors Sleeping outside the bed Dementia subtype Cultural differences Toilet behaviors Dementia subtype Sleep apnea Hawthorne effect	
	Data management	Management of data from a processing or governance perspective.		Patient access Societal benefit Data sharing across clinical services Data processing	

**Table 3:** Themes and subthemes generated from the semistructured interviews with primary care physicians.

Theme and subtheme			Definition		Common topics
**Prospective acceptability:** **considers what makes the sleep mat acceptable to clinicians, from functionality to clinical feasibility and consequences**					
	Feasibility, reliability, and validity	Reflects concerns regarding practical implementation and trustworthiness of using the sleep mat for dementia risk detection.		Complexity of sleep behavior External influences affecting data accuracy Feasibility of use in end users’ homes Behavioral changes due to Hawthorne effect Patient acceptance of the mat. Comparison with existing digital products.	
	Dementia risk detection	Refers to both patient and health care providers’ challenges in interpreting, communicating, and understanding dementia risk scores, and concerns regarding the potential outcomes of dementia risk screening.		Perception of risk Purpose of the risk score Communication of risk Desire for diagnosis Patient Choice Actionability Negative consequences for patients	
**Health care provision: c** **onsiders broader infrastructure and logistical elements needed for risk score successful deployment**					
	Current clinical practice	Refers to the existing landscape of how sleep and related digital data are managed in primary care.		Sleep concerns Sleep management Consideration of consumer digital products in decision-making	
	Service provision	Refers to logistical aspects of implementation, such as who, where, and when the dementia sleep risk score should be offered.		Service delivery Clinical timepoint Target population	
	Health care readiness and actionability	Refers to how equipped the current health care system is to act on the dementia sleep risk score.		Actionability of risk score Resource readiness Integration into current pathways	
	Requirements for implementation	Factors required to be met to ensure successful deployment of the dementia risk score.		Cost and value Trust and credibility	
**Practical considerations:** **focuses on how results will be presented and understood by both patients and clinicians**					
	Data visualization	How the report is presented and shared with end users.		Report preferences Comparison with other reports for chronic condition screening Sharing the report with patients	
	Clinical interpretation and decision-making	Requirements for the clinician to interpret the data and make informed decisions.		Need for training and guidance Meaningful metrics Identification of reasons for poor sleep or anomalies Standardized decision-making tool	

### Thematic Analysis: Potential End Users

#### Theme 1: Practical Use by End Users

##### Overview

The first theme was practical use of the device and its use in a dementia screening service, which has 2 subthemes: considerations for practical use and cost implications.

##### Considerations for Practical Use

Participants identified practical considerations for using the sleep mat in an optimal way. Commonly, they considered bed (eg, mattress type, electric blanket usage, and bed size) and design features (eg, size and robustness of the device), safety issues such as fire hazards, and the need for digital connectivity, such as access to Wi-Fi. They highlighted the need for technical support to set up the device, and reminders and prompts to ensure the device was switched on and collecting data during the required period.

I was just going to say, when you wrote ‘a patient would install it,’ in some ways it may be better to explicitly refer to ‘or family or friend’ because even though they probably can install it, it's a very simple thing, but I know my grandma, with dementia, would have a lot of insecurity. She would think she's done it wrong. She wouldn’t know where to put it.

G3P02, female, London

Participants felt that a longer time period than the recommended 3 months would be required and suggested multiple follow-up assessments (eg, once a year) to assess progression or identify new risk signs. Additionally, there was some interest in keeping the device as a permanent monitoring feature. These perspectives may reflect a shift towards personal responsibility and autonomy in managing one’s own health, alongside growing expectations of continuous, data-driven engagement with health and well-being data in the emerging digital landscape.

##### Cost Implications

Cost implications of the device were discussed from the perspective of the end user, such as the price of purchasing the device personally and the additional expense of running the device in one’s own home. There was some advocacy for receiving the device on prescription and having running costs supplemented by the health care services, which may reflect an expectation that such costs are the responsibility of a publicly funded health care system rather than the individual. Other participants expressed willingness to purchase the device for their own use, reflecting a broader interest in investing in their personal health. Differing views may be shaped by individuals’ socioeconomic status, influencing whether participants regarded cost as a barrier to participation.

It would be the same as if you had to pay to have a medical test done. In this country, we don’t. It may take that bit longer, or whatever, but everyone has the chance to get treatment. Whereas, if you’re having to pay for something, people put it off.

G2P01, male, Newcastle

There was also some consideration as to whether the benefit of the device and dementia screening service would benefit the health care service by reducing medication and care costs.

I suppose as well, if you’ve got doctors that realize that early intervention is going to be better for them, they're going to save money on their practice and they're going to save on medication in the long term as well. So, if they're sold on the fact that it’s going to make a difference, well that would be a big difference for the practice.

G1P05, female, Newcastle

#### Theme 2: Prospective Acceptability by End Users

##### Overview

The second theme was prospective acceptability of the device and its use in a dementia screening service, with 3 subthemes: general acceptability of the device, comparison to other digital products, and comparison to other clinical markers.

##### General Acceptability of the Device and Digital Technology

Overall, the device was viewed positively, considered “unobtrusive,” “easy,” and beneficial to wider society. Possible negative perceptions related mainly to the microphone feature due to perceived intrusion of privacy, and anxiety over the possible results.

Knowing the mat is underneath you, recording this data, might cause you anxiety in itself. Thinking, “If I move-”

G3P06, female, London

Familiarity with technology was cited as an important factor for the general acceptability of using the device. Participants highlighted willingness to have their sleep data recorded for use in health care services or to support health care research, but expressed distrust in having the data stored, accessed, and used by commercial organizations or third parties. Similarly, some participants cited distrust in technology generally as a barrier to use.

##### Comparison to Other Digital Products

The sleep mat was compared with other digital products such as smart watches and phone apps, particularly considering the comparative accuracy to determine sleep, the ability to capture napping behaviors outside bed, and the incentive to use the sleep mat rather than an existing product. Data about sleep can be provided from existing devices, and this information was of interest to participants. Participants considered the sleep mat an additional tool to use to observe their sleep behaviors, alongside existing devices, suggesting an inherent trust in familiar technologies already used to monitor health and well-being This trust appeared linked to perceptions of visibility and access to data, with devices providing continuous real-time feedback viewed as more reliable or desirable because one could actively observe and interpret their own data. Contrastingly, the sleep mat’s limited scope of only capturing in-bed activity reduced its perceived value, with concerns that it would miss napping behaviors in other locations (eg, an armchair) and not provide an accurate report to clinicians.

There are lots of apps that track, for example, when you went to bed, when you woke up, and then they can do your REM cycles. How would you incentivize someone to see the value of putting a device under their bed when they could actually change nothing and still get that data for themselves?

G3P02, female, London

##### Comparison to Other Clinical Markers

Some participants suggested a preference for the sleep mat over other clinical tests they were aware of or had experience of, such as cognitive interviews, stool samples, blood tests, and sleep diaries, as it is less obtrusive and stigmatizing. They felt it could be paired with other tests to support screening efforts, suggesting a desire for a holistic screening service.

I can see the benefits of the mat if it’s used in conjunction with other tests, rather than being a sole determinant.

G3P01, male, London

#### Theme 3: Clinical Management

##### Overview

The third theme was clinical management, largely focusing on the potential dementia screening service proposed, encompassing 3 subthemes: service provision, reciprocal communication, and postscreening actions.

##### Service Provision

Participants discussed who the dementia risk detection service would be provided to in terms of age, family history, and existing health conditions, and at which clinical stage. They suggested that the service might be provided when people present with sleep problems, and that it should be considered an early detection or preventative service. There were mixed feelings regarding the provision of the service to those presenting with memory problems, generally feeling this was too late, although one participant thought it could be provided to people with mild cognitive impairment.

One of my initial thoughts was, by the time you’ve got to the memory clinic, very often that almost seems too late.

G1P01, female, Newcastle

Participants also commonly advocated the provision of the sleep mat and service to those with a family history of dementia. They felt that younger people may be more inclined to use the service and make risk-reducing lifestyle changes accordingly, although this may be deterred by the perception that dementia is “for old people” (G1P03, male, Newcastle). Additionally, provisions for middle-aged people and sports players were mentioned as these groups were perceived to have an increased risk of dementia.

Participants also suggested that the sleep mat could be used for additional purposes, such as monitoring sleep in those with concerns, acting as a safety device for people with dementia (eg, detecting falls), as a general well-being device, or as a method to monitor disease progression in dementia.

##### Reciprocal Communication

The communication of data and results was discussed by participants. Participants wanted to be able to see their own data, while expressing the need to have an expert relay what that data means to them.

Well, really, I mean, the common lay person doesn’t know what information they’re looking for, really. I mean, at the end of the day, it seems, sleep, they don’t know the in depths of it, what’s good and what’s bad. They’ll be looking for the GP or the professional to let them know the information that they thought was important.

G2P02, male, Newcastle

Participants also expressed the desire to be able to bring results from the sleep mat to the doctor if they were worried before the 3 months were up, or to be able to use the data to communicate abnormalities with clinicians themselves. This reflects the interest in actively engaging with health care services through control of one’s own data, rather than passively waiting for the clinician’s decision.

##### Postscreening Actions

Participants expressed the need for clinical support or self-management avenues following sleep-based dementia screening, particularly regarding improving their sleep. Referrals to sleep specialists, talking therapies, and cognitive behavioral therapies were proposed. Some participants expressed willingness to adopt lifestyle changes around sleep if they received a high-risk score or demonstrated poor sleep.

"We don’t say you’re going to have dementia, but we think you’re not sleeping very well.” Whether I’m going to have dementia or not, I’m going to try and start looking at things that will help me to sleep better.

G4P02, female, London

#### Theme 4: Data Concerns by End Users

##### Overview

The final theme identified was data concerns, composed of 2 subthemes: data accuracy and management.

##### Data Accuracy

Participants highlighted multiple factors that could potentially impact sleep and the DRI-SI results, including alcohol, erratic sleep patterns, medications, mental health, daytime sleep outside of the bed, dementia subtypes, and sleep apnea. Additionally, participants outlined concerns regarding characteristics or experiences which might inaccurately be perceived as sleep behaviors relating to dementia risk, such as the influence of bed partners (eg, waking up when partner gets up for the toilet) or incorrectly detecting a bed partner’s heart rate as their own including spouses, partners, children and pets; behaviors in the bed besides sleeping such as watching TV or intimate moments; and frequent nighttime toilet use. They were concerned that the microphone features may inaccurately classify white noise or talking as sleep-related noises. Finally, they highlighted that results may be impacted by the accessibility of the device; for example, only collecting and comparing data from those who could afford to use the device. Multiple participants also suggested that end users may change their sleeping behaviors due to a heightened awareness of sleep monitoring.

What would be disastrous is if you had this thing and then you stopped doing whatever it is you’re doing or trying to change whatever it is, the way you normally do things because you’ve got this mat.

G4P02, female, Newcastle

##### Data Management

Participants generally expressed the desire to have access to their own sleep data as collected by the device. Some participants considered wider access to their data, such as by researchers, as acceptable if it was positively benefiting wider society, and advocated for the data to be shared across clinical services.

So long as it’s anonymized, I don’t have a problem with the collection of data. Also, provided that it’s doing useful research, but also that the research is open, again, I wouldn’t mind. I wouldn’t want it being sold to the highest bidder… I wouldn’t want them being sold to a private company to make a profit off. I’d want any medicine that comes out of it to be open.

G2P01, male, Newcastle

Participants questioned who would be processing the sleep data and suggested that it should be processed by the health care services rather than third-party organizations. These perceptions likely reflect wider social anxieties about data misuse and commercial exploitation, and limited awareness of how health data is often managed through the commissioning of external companies to process tests and support digital infrastructure.

But I’m saying that, if you’re saying, from a patient point of view, “Where’s my data being held and who’s processing it?” I would want it to be processed- I don’t see why, in the medium to long term- In the short term it’s different. But in the medium to long term, why can’t that be processed within the auspices of the NHS?

G4P01, male, London

### Thematic Analysis: Primary Care Physicians

#### Theme 1: Prospective Acceptability

##### Overview

Physicians considered what makes the digital sleep mat acceptable to health care professionals, from functionality and validity of the device to clinical feasibility and consequences of dementia risk detection. Two subthemes were identified as follows: (1) feasibility, reliability, and validity and (2) dementia risk detection.

##### Feasibility, Reliability, and Validity

Regarding the use of the device, physicians considered how feasible and acceptable it would be for use in potential end users. They generally considered it noninvasive and simple, although one mentioned that there may be concerns regarding data privacy. They wanted to understand the benefit of the digital sleep mat in comparison to other familiar digital devices, such as smart watches, particularly as the digital sleep mat may not detect napping behaviors. There were some practical concerns about end users not using it correctly or unplugging the device.

I suppose the cables dangling around and could that get in the way? Are people just going to say, “Oh that’s in the way,” or trip over it and remove the sensor.

GP4

Physicians highlighted sleep as a complex, multifactorial behavior, with many different confounding factors for poor sleep quality, including nighttime toileting, partners’ sleep behaviors, low mood, chronic pain, heart problems, and sleep apnea. They felt it would be difficult to disentangle these factors from the DRI-SI and required more information on how these confounders would be accounted for. Most physicians agreed that there may be a Hawthorne effect early in the sleep monitoring process, whereby end users would change their sleeping behaviors due to a heightened awareness of being monitored, but that the 3-month period would alleviate this effect.

There’s so much more about sleep than just… yeah, and I think that would be harder to sell to patients and clinicians. They’d be like, “Well, actually there’s so much more to sleep than just memory and the picking up dementia.”

GP2

Whenever a patient knows they’re being monitored for something, I suspect what they would do is they would try to be a good, model patient. They’ll go to sleep on time and they’ll try to make sure they’re sleeping above the sensor. But I think the fact that it’s over three months means that your initial data might seem quite pure, let’s say. But then, later on, maybe after the first few weeks, when the patient has got used to it, they may have forgotten about the sensor. So, then, it becomes a much more natural version of their routine.

GP1

Additionally, physicians wanted information about the scientific validity of the device and DRI-SI, including the validity of sleep as a risk marker of dementia, the sensitivity and specificity of the score to detect dementia risk, at which clinical stage or disease subtype, and the measurement error and reliability of the device.

And that kind of comes into, is poor sleep a symptom of you’re going to develop dementia, or is it a risk factor that causes you to develop dementia?

GP7

I just want to be knowing how it’s working and how accurate it is.

GP4

##### Dementia Risk Detection

Physicians described being familiar with communicating risk scores for other clinical conditions, such as diabetes and heart disease, and generally felt confident that patients understand these concepts. However, they noted that fear and stigma of dementia and ageing stereotypes may reduce patients’ desire to know their dementia risk or to understand the role sleep may play in it, although this appears to be shifting for the younger older generation. They noted that some patients may struggle to separate risk from diagnosis and may consider the condition as an inevitability. This may be due to ineffective communication, as explaining risk requires skill and experience.

Sometimes people will have experience of dementia and older parents or older relatives, and they'll have almost decided… Well, both my parents had this, you know. So, I'm going to get this. So, I don't know if the score per se would be but in terms of whether they would see that as a modifiable thing, in the same way that they would look at their blood pressure, their cholesterol. I'm not sure.

GP3

What they understand is the feelings that those numbers trigger off in them. So, in my experience, when I explain to someone that, “You might have a 20% or 30% risk of having a stroke,” or whatever, the patient doesn't hear 20% or 30%. What they hear is, “Stroke,” so that they hear the thing that they can latch onto, which is the thing which then triggers off an emotional response in them.

GP6

To support discussions regarding dementia risk, physicians highlighted that they would need to understand the relationship between sleep and dementia, the components of the risk score, and have guidance on how to explain it to patients, as is the protocol with other common screening processes.

I think the one thing that they would want to know is, what is the risk of a sleep disturbance? Or what is it that makes sleep something that puts you at risk of dementia?

GP1

Finally, physicians considered the provision of the DRI-SI as acceptable only if there was a consequential positive action to improve health or reduce and modify risk. Without this, physicians expressed concerns about frightening patients and causing unintended harm by sharing a risk score without a pathway to modification.

I wouldn’t share it with a patient and frighten them and say that “You’re moderate risk of dementia,” because I’m sorry until somebody can explain to me the clear evidence for that then I would cause harm to someone by doing that so…

GP2

Potential interventions need to be carefully considered and tailored to a patient’s individualized response, as this information may support some to make lifestyle changes, but negatively impact others due to emotional connotations and comorbid conditions. Some physicians questioned the need for the DRI-SI based on the current risk reduction evidence, citing primary population-level prevention as a simpler, effective strategy. However, risk modification was not the only considered benefit. Risk detection could also support advanced care planning and trigger safety assessments, such as reviewing driving abilities. Additionally, physicians wanted to know if the DRI-SI could assess intervention effectiveness, with a reduced score showing modified risk.

There's a possibility and there's a plausible scenario, given that, in my opinion, a lot of it is driven by emotion triggers, that the people that are least well equipped to be able to actually change their behavior, because their response gets driven by an overwhelming emotional response, then end up not being able to change their behavior, even if they are, in fact, at the greatest risk of getting dementia.

GP6

#### Theme 2: Health Care Provision

##### Overview

Physicians considered the broader infrastructure and logistical elements required to successfully deploy the digital sleep mat and subsequent DRI-SI within health care, leading to four subthemes: (1) current clinical practice, (2) health care readiness and actionability, (3) service provision, and (4) requirements for implementation.

##### Current Clinical Practice

Physicians indicated that they often speak to people about sleep problems, usually part of a larger consultation. However, sleep concerns do not usually occur in the context of help-seeking for dementia-related symptoms. Physicians talked about a variety of sleep management techniques they might use, often involving identifying the root cause and trying to make small changes to alleviate the impact. They indicated that they would generally refer people to social prescribers to speak about sleep hygiene, or to websites and apps to encourage lifestyle modifications rather than medical treatment. However, physicians felt that sleep management is variable depending on the clinician and their experience, and generally indicated that sleep is not an area where they have great clinical expertise.

Well, I don’t have any treatments for poor-quality sleep. And I have asked the powers that be, and I’m not convinced that there’s anything that I can really do. So, we talk about sleep hygiene, and exercise, and not eating too late at night, and limiting caffeine intake, particularly in the latter part of the day. Of course, we do all that. We have a speculative idea of what melatonin might do. And many people just go buy it. We avoid benzodiazepines, in the form of hypnotics, very firmly, but we do give them. If there’s complete sleeplessness, usually, on a short-term basis. But what’s a treatment for sleep disturbance?

GP5

I don’t have any formal training in sleep stuff I suppose like this so I’m coming back to myself.

GP4

Physicians also indicated that an increasing number of patients were bringing their own direct-to-consumer devices and outputs relating to sleep, physical activity, and heart rate to a consultation, but there were mixed views on what to do with this data. Some physicians appreciated the role these devices can play in empowering patients and promoting self-advocacy, while others felt that they would not know how to interpret or use the data, and do not allow it to impact clinical decision-making.

I think it's a great thing. I think, if people are interested and engaged their health, then that's fantastic, isn't it? But I also think, yeah, it's quite interesting to look through someone's app or their resting heart rate.

GP6

It wouldn’t be like the main point of a consultation if anything… And actually, I might note it down and stuff but it doesn’t add much to the history of the condition because I don’t then go, “Okay. So, he’s had this much REM sleep or this much non-REM sleep.”

GP1

##### Health Care Readiness and Actionability

A common area of discussion amongst physicians regarding the implementation of a dementia risk detection service based on the digital sleep mat was a lack of resources and overburden on primary care. Physicians were generally concerned with their lack of time and “the never-ending list of things that the GP does” (GP7). They felt that conducting the proposed dementia risk detection service would remove resources from other critical tasks, such as annual reviews, cognitive assessments, and in-depth conversations about memory concerns. They also discussed how detecting dementia risk might lead to an influx of people being screened, leaving fewer resources for those who require postdiagnostic support and care.

Because you haven’t got… you’ve got 10 minutes with someone and I’m looking to see if you’ve got a risk of dementia. I’m not going to be going on about sleep for 10 minutes, I’ll be looking at function and cardiovascular risk and all this else.

GP2

We already know that many people, when they are diagnosed, experience a high degree of abandonment and not really getting very much post-diagnostic support at all. So, the problem is, quite understandably, many people are really interested in trying to widen the funnel and get more people in at an earlier stage and get something done about it. But the problem is that that means, unless you have an extremely effective filter and funnel at a relatively early stage in the funnel, you have more people progressing through.

GP6

Physicians wanted to know how the service would integrate into current care pathways and have clear guidance on where to refer high-risk individuals.

“Here, by the way, here’s the risk for dementia, and it's really high,” there has to be a, “So what?” a, “So what? It's really high. What can I do that is going to change that?” and in a way which is personalized to the individual, because otherwise referring back to the GP will mean diddly-squat.

GP6

##### Service Provision

Physicians discussed where this sleep-based dementia risk detection service would best fit and which population might benefit the most from it. Generally, they felt it should be commissioned as part of the NHS as opposed to a direct-to-consumer service, citing clinicians’ “conscious incompetence” with unfamiliar data, exacerbating health inequalities, and over-investigation due to the uncontrolled provision of the digital sleep mat.

So, I think that that kind of digital health revolution is going to create significant problems going forward. The sleep mat isn't going to contribute to that if it's done in a controlled way and it's monitored and it's used in the right people and the right population.

GP7

Within the NHS, physicians advocated for the integration of the digital sleep mat into either established health check services or annual reviews, or to consider the DRI-SI as one marker in a clinical toolkit for a dedicated dementia screening service. Regarding the target population, patients with existing risk factors such as diabetes and heart disease were commonly mentioned, alongside people with a family history of dementia, genetic vulnerability, or middle-aged people who chose to be screened due to the need for future planning, such as caregivers for disabled children or partners. Some physicians also suggested using the DRI-SI at a later clinical time point, such as in people with suspected dementia or mild cognitive impairment, and to increase rates of diagnosis within the care home population.

You know, everybody who has heart disease, diabetes, that kind of thing they get, you know, anywhere between… sort of… one and thee review appointments a year with the practice nurse. And again, I think the opportunity to have a sleep mat would fit quite nicely into that.

GP3

If this was part of a wider NHS Dementia Health Check, I think it could certainly fit in and I think that service is massively needed and I think it would be a hugely beneficial use of primary care's time.

GP7

##### Requirements for Implementation

To have this service integrated into the NHS, physicians highlighted the device and DRI-SI to be trustworthy and credible, as evidenced by empirical evidence published in peer-reviewed journals, guidelines from credible sources, such as the National Institute of Clinical Excellence, and appropriate licensing, such as acquiring a CE mark for this use. Additionally, they felt that the cost-benefit must be apparent and that the service would need to be funded with financial incentives for service providers.

I think you just find that the reality for any practice would be that unless there was some funding tagged onto it that they could use, they wouldn't.

GP3

#### Theme 3: Practical Considerations

##### Overview

**Figure 1:**
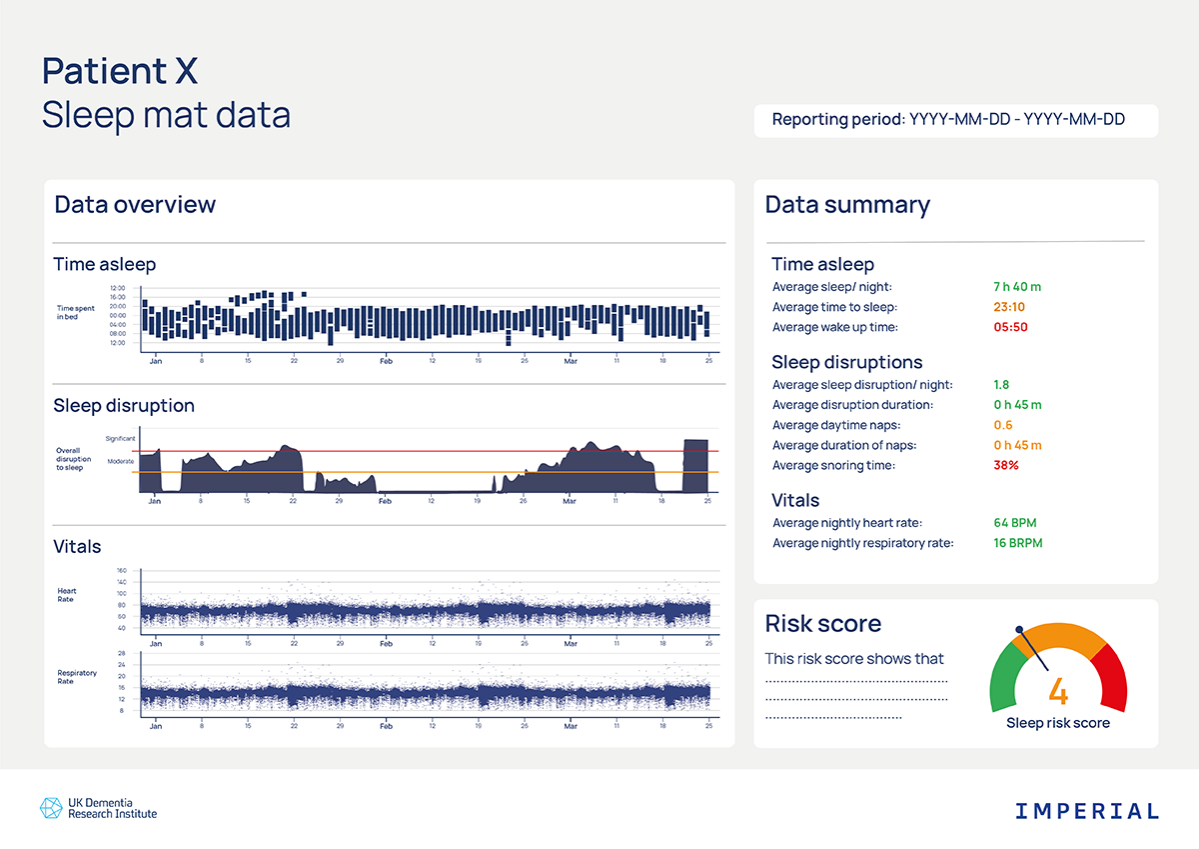
Example prototype of the data visualization of the Dementia Research Institute Sleep Index risk score.

The final theme focuses on how results will be presented and understood by both patients and physicians, involving two subthemes as follows: (1) digital sleep data report, and (2) clinical interpretation and decision-making. These data were largely derived from discussions with the physicians after presenting them with the prototype clinical report (Figure 1).

##### Digital Sleep Data Report

Generally, physicians felt the digital sleep data report was “easy to read,” “presented nice and clear,” “helpfully color coded,” and “simpler” than other reports. They tended to be drawn towards the graphs. They compared the report to other clinical reports that they were familiar with, such as echocardiograms, DEXA scans (ie, a bone density test), or QRISK results (ie, a risk score for developing cardiovascular disease), highlighting that these outcomes generally have a standardized summary to aid interpretation and support clinical decision-making. Physicians felt more comfortable with this standardized reporting approach as “you know what is going to come next and you know which bits to focus on” (GP1). They mostly felt comfortable with sharing this report with patients but highlighted that they would need guidance and a “patient decision aid document.”

But in terms of the presentation, it's very clear in terms of the science for me to understand, but also for me to communicate that to a patient; it's, it's very, very good, I think.

GP7

##### Clinical Interpretation and Decision-Making

Regarding their use of the report to make clinical decisions, physicians highlighted the need for meaningful metrics. They commonly focused on the vital signs in the report, due to their greater familiarity with interpreting and communicating these results. Some tried to interpret the sleep scores, such as sleep disruption, but highlighted the need for training to use such metrics clinically. Physicians also felt that some patient feedback or self-reporting would support their decision-making when considering anomalies in the data. Finally, they wanted a standardized decision-making section within the report to guide them on what actions to take next with the patient.

In clinical practice we’re less attuned to looking at sleep, the sleep graph, so my immediate attention would be to the vital signs because then that’s data that we’re more familiar in interpreting.

GP4

I think a summary by the technician who’s reporting this, to say, “These are the things it would be helpful to counsel the patient on,” would make our job a lot easier. Because, otherwise, if this is going to be used nationally, you’re going to have to train a lot of GPs to be able to determine that. It would take a lot of effort to do that.

GP1

## Discussion

### Principal Results

This is the first study to capture perceptions and attitudes of potential end users and primary care physicians regarding the use of a digital sleep mat to derive a digital sleep biomarker (DRI-SI) for dementia risk detection. Potential end users find both the device and the proposal of dementia risk detection generally acceptable; their concerns focused on the practicality of the device, their trust in technology, and the personal impact of sleep monitoring and dementia risk detection. In contrast, primary care physicians were reluctant to provide the device or engage in dementia risk detection without clear guidelines, empirical evidence of benefit, and clinical actionability. Findings from this study are informative for the development and implementation of other noninvasive biomarkers for dementia, particularly those using digital methods.

### Comparison With Prior Work

End users and primary care physicians differed in their views regarding the acceptability of the use and implementation of the DRI-SI. Potential end users largely focused on the use of the digital sleep mat and the information it could provide, considering how it would fit into their daily lives and how they felt about continuous data monitoring. Regarding dementia risk detection, they believed that dementia risk reduction techniques could be implemented early and effectively, reducing overall health care costs and burden. These findings reflect recent systematic reviews and surveys showing positive attitudes of the general public around dementia risk detection due to beliefs of positive outcomes, such as slowing the disease via lifestyle changes and providing more time to plan for the future [27,28]. Comparatively, primary care physicians were more concerned with the clinical validation and health care system integration of the DRI-SI, requiring a more robust evidence base for implementation of a digital sleep biomarker for dementia. This echoes a recent systematic review, which reported that primary care physicians were reluctant to consider the provision of dementia risk detection, due to a lack of knowledge, skills, and training on dementia, resources to provide the service, and concerns regarding the consequences to patients [29]. While physicians held concerns regarding the accuracy of the DRI-SI to detect dementia risk, they also highlighted the more general issues of limited evidence regarding benefits versus harms of dementia risk detection and for accessible treatments that can slow or stop disease progression [25,38-41]. The complexity of the issue moves beyond the development and validation of the digital biomarker and highlights the need for good-quality evidence on clinical actionability of dementia risk detection and benefit to patients and the wider health care system.

### Strengths and Limitations

Strengths of this study include the recruitment of potential end users with a range of ages and experiences related to dementia (ie, care partner, family history, subjective memory concerns), a high representation of males, with ethnic diversity representative of the United Kingdom’s 2021 census [42]. Physicians were recruited from across different clinical practices and English regions, providing a greater diversity of professional experiences. Physician-focused interview guides were informed by the end user perspectives, reflecting real-world concerns, and supporting alignment of stakeholder perspectives across the proposed clinical pathway. Finally, throughout this substudy, we have engaged meaningfully with people with lived experience of dementia through our focus groups with potential end users and our partnership with public patient involvement contributors. To address recently reported gaps in meaningful end user engagement in digital health research for neurological populations, we have reported these efforts in adherence to the recommended minimum reporting guidelines [43].

Clinical viewpoints in this study are limited to primary care physicians; this decision was based on the perspectives from end users of where the DRI-SI would best fit. However, this limits our understanding of the broader clinical perspective of those involved in the dementia diagnosis and care pathways, such as old age psychiatrists, geriatricians, and neurologists. Additionally, perspectives are limited to end users and physicians living in England and may differ internationally. For example, in the USA, the American Academy of Neurology and the Alzheimer’s Association both recommend annual cognitive screening for older adults [44,45], which may lead to greater willingness to uptake the DRI-SI as a dementia risk detection tool. We also did not collect detailed demographic data on end users, such as education level or socioeconomic status, so it is unclear whether the participants included reflected the established dementia risk factors [3].

### Implications for Policy and Practice

Digital biomarker efforts often propose value as population-wide dementia risk detection tools, as they are unobtrusive, objective, usually passive, may reduce health care burden by being conducted remotely, and are speculated to detect dementia risk in “real time” as they continuously monitor changes in physiology and behavior [4,10,22,23,26,46]. However, the reluctance from physicians in this study to use the DRI-SI without its integration in clinical guidelines reflects the wider controversy around dementia risk detection [26,40]. No detection tool, digital or otherwise, has yet to meet the internationally-recognized criteria for population screening, which require an adequate understanding of the latent stages of the condition, an accurate and acceptable tool for both patients and physicians, a target population, provision of an accepted treatment, reasonable facilities for diagnosis and treatment, and economic cost-benefit [8,10]. As such, the UK’s National Screening Committee currently recommends against such programs [26]. Many of these criteria go beyond the scope of digital biomarker developers’ ambitions and require a comprehensive multidisciplinary team science approach, which would include physicians, health care commissioners, and patient voices. Despite these significant barriers, the physicians in this study felt strongly that, if implemented, the DRI-SI and resulting dementia risk detection service should be integrated into existing clinical pathways with guidance for referral in the national health care services.

To meet this challenge, the emergence of “brain health” clinics in the United Kingdom could offer a potential bridge to continue to gather the required evidence while working with health care providers. Brain health clinics are joint clinical-research services that provide access to high-quality assessments not routinely available to patients under investigation for cognitive impairment and physicians, including biomarkers under investigation [47,48]. Recent reports show that 93.5% of patients consent to the use of their data collected as part of the brain health clinic to be used for research [48]. Brain health clinics are considered a safe and acceptable care model for the conduct of more novel biomarker assessments, and have the resources to implement secondary prevention interventions for high-risk individuals [49]. They also meet the suggestion by both end users and physicians to implement the DRI-SI as part of a wider clinical toolkit and dedicated service to alleviate burden from primary care, while allowing integration into an already established clinical pathway with actionable endpoints, that is, dissemination of information to clinicians in the memory clinic [29,48]. The term “brain health” can also be less stigmatizing than “dementia” and is preferred by primary care physicians when making referrals [50]. Further work should consider capturing the attitudes and perspectives of brain health clinic staff and associated memory clinics regarding the use of the DRI-SI to support dementia risk detection.

### Looking to the Future: Wider Impact for Digital Biomarker Research

#### Overview

While the optimal application of the DRI-SI, like most digital biomarkers, remains unclear, further work could develop and validate it for a clinical service, as previously proposed, or as a tool to support identification of participants for clinical trials and disease-modifying targets [10,26]. From our findings, we have identified 5 key recommendations that should be considered for all proposed applications for the DRI-SI and are relevant to digital biomarkers more widely, prior to any integration into health care systems.

#### Clinical Staff Require Training on the Proposed Physiology or Behavior

In both this study and the wider literature, physicians indicated limited knowledge and education on dementia-related risks, particularly the association between sleep and cognitive impairment [29]. This leads to mistrust in the DRI-SI, a lack of confidence in how to explain this link to patients, and heterogeneous intervention strategies. Any digital biomarker will require clear and concise clinical information on the underlying association with dementia, which can be shared with end users.

#### Digital Technologies Need to Be Usable for All Stakeholders

Both end users and physicians held concerns about the usability of the device. End users were primarily concerned about setting up the device and ensuring its continued maintenance, while physicians felt that setup and maintenance would be beyond their resource abilities. Digital biomarker developers need to develop sustainable models of technical support, should these devices be integrated into clinical services or trials. This should include tailored support for individuals most at risk of digital exclusion, to reduce further exacerbation of health inequities in these groups [30,51-53].

#### Technical and Clinical Validity Need to Be Clearly Communicated

Both end users and physicians worried about confounding factors influencing data accuracy. Previous research regarding clinical use of artificial intelligence models reports that physicians are more likely to trust these tools if they can readily explain and justify them; this requirement was highlighted by both our stakeholder groups [54]. Digital biomarker developers need to develop adaptive algorithms that account for such factors and communicate clearly with physicians how these algorithms work so that they can answer concerns from end users. Additionally, physicians need access to evidence of clinical validation via credible sources (eg, National Institute of Clinical Excellence guidelines and peer-reviewed journals) to improve confidence in the digital biomarker. Additionally, both stakeholder groups indicated a rise in the desire of patients to manage and advocate for their health and well-being, with an increased ability to continuously monitor relevant outcomes through “wellness” consumer-based products such as smart watches. However, both groups also highlighted a lack of “expert” understanding of these data and the continued need for clinical interpretation to make sense of digital health metrics. While self-management supported through digital means is a key principle of the NHS’ 10 Year Health Plan for England [55], there is a disparity between the standards of evidence required for commercial “wellness” devices and those approved for clinical use. Clearer communication of these differences to the general public and clinical bodies is essential to prevent unnecessary anxiety among the “worried well” and to support clinicians with reliable, evidence-based guidance on trusted digital tools.

#### Outcomes Need to Be Meaningful and Interpretable

End users and physicians wanted to understand and see easily interpretable data with meaningful outcomes, that is, aspects of health that have personal importance for end users [56]. This is not just important to our stakeholders; evidence of meaningfulness of outcomes is now required by regulatory bodies such as the European Medical Association and the Food and Drug Administration for qualification of clinical endpoints [57]. Additionally, patient decision aids and clinical results with standardized advice need to be provided through effective data visualization tools, codeveloped with physicians and end users to ensure effective communication and decision-making, as seen with common screening tools such as the QRISK [58].

#### Transparency in Data Management and Governance

Aligned with common perspectives on digital technologies, end users indicated that they were concerned with their data management and governance, preferring their data to be held and processed by trusted health services rather than third-party providers [26]. To improve trust in these processes and meet recent ethical guidance, digital biomarker developers should be transparent about who has their data and for what use, with guidance on how physicians can discuss this with end users [59,60].

### Conclusions

Overall, this study found contrasting perspectives regarding the acceptability of using a digital sleep biomarker as a dementia risk detection tool, based on the current evidence base and clinical practice. Further work to integrate the DRI-SI in clinical-research pathways may support continued development and improve clinical acceptability and adoptability. Findings from this study support broader recommendations for the development of digital biomarkers for dementia populations, including requirements of further training for clinical staff, development of usable technology and meaningful digital outcomes, and transparency regarding technical and clinical validation and data governance procedures.

## Data Availability

The datasets generated or analyzed during this study are available from the corresponding author on reasonable request

## Appendix

Multimedia Appendix 1 Topic guide for focus groups.

Multimedia Appendix 2 Interview Schedule for primary care physicians.

## Abbreviations

DRI-SI: Dementia Research Institute Sleep Index
NHS: National Health Service
NIHR: National Institute for Health Research
