# Supplementary material for: End User and Primary Care Physicians’ Perspectives on Digital Innovations in Dementia Risk Detection: Focus on a Digital Sleep Biomarker
Source: JMIR Aging. 2025 Dec 1;8:e74307. doi: 10.2196/74307 (PMC12706451; doi:10.2196/74307)
Supplement: Multimedia Appendix 1 [file aging_v8i1e74307_app1.docx]

**Appendix 1: Topic guide for focus groups**

**Study introduction** (Research team delivering presentation) - 15 minutes

- Explanation of the InSleep46 study, including the interventional arm, and the Dementia Research Institute Sleep Index biomarker.
- Explanation of the public and professional consultation project
- Demonstration of the Sleep mat and how it works.

**Presentation –** 10 minutes

**Illustration of a hypothetical diagnostic service**

- Presentation of what a diagnostic service based on sleep data might look like:
- Person approaches GP or memory clinic with concerns about memory
- Standard cognitive tasks carried out in consultation
- Patient returns home and receives sleep sensor in the post
- Patient installs sensor and uses it for 3 months.
- Patient returns to GP / Memory clinic for follow up consultation
- Sleep data is presented and discussed as one of several factors indicating that the person may or may not have dementia.

**Group discussion –** (1-2 hours with breaks)

**Questions for group discussion in response to the presentation:**

**Worksheet 1 practical issues (approx. 20 mins)**

Are there any practical problems you would have in using a sleep mat under your mattress for 3 months?

- If you often sleep badly, would this service cause you any increased anxiety? (about sleep or dementia risk)
- Would you trust the mat to collect accurate information about your sleep?
- Would you be prepared to keep a sleep diary, of how you felt you slept during this period (in an App / on paper?)

**Worksheet 2 – Outcomes – (approx. 20 mins)**

**Facilitate discussion about how people would feel if they were given a mat to use to identify their risk eg:**

- To what extent did you think that sleep patterns could be used to help with a dementia diagnosis?
- How would you feel about using a sleep mat for 3 months as part of a diagnosis procedure?
- Would you use this service if your GP said:
- I am concerned you might have dementia and want more information
- I do not think you have dementia, but this service will help me be more certain
- Its unlikely you have dementia, but using this service might give you more peace of mind.
- If after 3 months, how would you feel if the GP said:
- Your sleep data is indicating that you may have dementia
- Your sleep data is indicating that you don’t have dementia, despite your other concerns
- Your sleep data so far is not very helpful or conclusive, please could you keep using it and come back in another 3 months?
- Your sleep data is inconclusive, so we can only use the cognitive tests to inform a diagnosis.

**Worksheet 3 - Information sharing (Approx 20 mins – or less depending on what time allows**

- What information would you want or expect during the three months?
- How would you want to receive this information.
- What information (if any) would you want before going to the follow up appointment?
- With what you have seen so far, do you have any ideas how we can make this service more appealing, or more useful to people?

**Other questions**

- What questions do you have about how such a service might work?
- If it wasn’t available on the NHS, would you pay for such a service? What would be an acceptable cost to the user?
