# Supplementary material for: End User and Primary Care Physicians’ Perspectives on Digital Innovations in Dementia Risk Detection: Focus on a Digital Sleep Biomarker
Source: JMIR Aging. 2025 Dec 1;8:e74307. doi: 10.2196/74307 (PMC12706451; doi:10.2196/74307)
Supplement: Multimedia Appendix 2 [file aging_v8i1e74307_app2.docx]

**Appendix 2: Interview Schedule for primary care physicians**

1. **Risk vs Diagnosis**

The sleep mat data would not be sufficient to provide a diagnosis and would only

provide a ‘risk score’ associated with likelihood of dementia. How do you think

healthcare professionals, patients and families would relate to and understand this

idea?

2. **Trust in data**

The sleep mat is located under the mattress on the side of the bed of the patient for

a period of 3 months. The patient does not need to do anything other than position

the device, plug it into a power supply and follow their normal routine. To what extent

would you trust the resulting outputs to accurately reflect the sleep of the associated

participant?

*Prompts*

*- what factors might increase of decrease you trust in this data?*

*- do you think patients are likely ‘game’ the process at all? In what way?*

**3. Identifying the most suitable patients.**

What are your thoughts on how we should identify and reach the patients who would

benefit from this most, considering that ideally this should be considered earlier than

current diagnostic methods?

*Prompts*

-How would you prioritise patient selection?

4. **Commissioning** – Could you see this solution as an NHS provided service, and/or a

consumer purchased service? (for example, cancer screening v.s. Zoe gut health

app).

*Prompts*

*- If NHS – who would be best placed to provide the devices, and act on the results?*

*- If consumer - how would this effect how Healthcare professionals regard the output*

*advice from the service?*

5. **Duty of Care**

Poor quality sleep, whatever the cause, is a contributing factor in the assessment. If the

sleep assessment demonstrates that the patient has poor quality sleep, how might

this be clinically managed?

*Prompts*

*- Would clinical management of poor quality sleep change if the patient said their sleep*

*quality was poor due to an external influence, such as as a result of their partner’s*

*health needs or due to a side effect of medication? If so, how might this work?*

*- Would your interpretation of the dementia risk be affected by the patient saying their*

*sleep quality was poor due to an external influence?*

**6. Feedback on Prototype**

(show image of prototype report).

Here is a prototype of what a report on the assessment after 3 months might look

like. Could you please describe what you see?

Is there anything you don’t understand?

Is there anything missing that you would like to see?

Do you have any other suggestions or comments?

(Also ask to share prototype report for feedback by email after the interview, allowing

for a bit more consideration of the detail..)

**7. Wrap up**

Thank you, that is all our, unless you have any other thoughts of comments about

this?
